# State-of-Charge Estimation of Lithium-Ion Batteries Based on the CNN-Bi-LSTM-AM Model Under Low-Temperature Environments

**DOI:** 10.3390/s26010264

**Published:** 2026-01-01

**Authors:** Ran Li, Yiming Hao, Mingze Zhang, Yanling Lv

**Affiliations:** 1 Engineering Research Center of Automotive Electronics Drive Control and System Integration, Ministry of Education, Harbin University of Science and Technology, Harbin 150080, China; 2 School of Electrical and Electronic Engineering, Harbin University of Science and Technology, Harbin 150080, China; hym0933@163.com (Y.H.); mzzhang@hrbust.edu.cn (M.Z.); yanling0828@hrbust.edu.cn (Y.L.)

**Keywords:** SOC estimation, CNN, Bi-LSTM, attention mechanism, low temperature environment

## Abstract

Accurate state-of-charge (SOC) estimation is essential for lithium-ion battery management, especially under low temperatures where traditional methods suffer from noise sensitivity and nonlinear dynamics. In this paper, a hybrid deep learning model integrating a one-dimensional convolutional neural network (1D-CNN), bidirectional long short-term memory (Bi-LSTM), and an attention mechanism (AM) is introduced to enhance SOC estimation accuracy. The 1D-CNN extracts local features from voltage and current sequences, while Bi-LSTM captures bidirectional temporal dependencies, and the AM dynamically emphasizes critical time steps. Experiments conducted on the Panasonic 18650PF dataset at temperatures ranging from −20 to 0 degrees Celsius show that the proposed CNN-Bi-LSTM-AM model achieves a mean absolute error (MAE) of 0.17–0.77% and a root mean square error (RMSE) of 0.33–0.94% under US06 and UDDS driving cycles, outperforming CNN-LSTM and CNN-Bi-LSTM benchmarks. The results demonstrate that the model effectively handles voltage distortion and nonlinearities in low-temperature environments, offering a reliable solution for battery management systems operating under extreme conditions.

## 1. Introduction

Lithium-ion batteries are widely used in electric vehicles and energy storage systems because of their high energy density and long cycle life. In the battery management system (BMS), the SOC and the state of health (SOH) are two pivotal indicators for system performance and durability management [1,2]. Improving the accuracy and stability of the SOC estimation algorithm is of great significance to prevent overcharging and overdischarging, extend the service life of battery cells, and enhance charging and discharging efficiency [3,4]. Presently, global lithium-ion battery SOC estimation methodologies are typically classified into three primary categories: the direct measurement method, the model-based method, and the data-driven method.

The direct measurement method primarily determines the battery’s SOC by analyzing on-site collected data. The coulomb counting method calculates the SOC by accumulating current over a period, effectively tracking charge and discharge cycles. However, the system operates as an open-loop mechanism, making it particularly prone to inaccuracies due to imprecise initial SOC assessments and inaccuracies in the current measurements [5]. The open-circuit voltage method (OCV) is a frequently applied direct SOC estimation procedure. Wang et al. [6] proposed an open-circuit voltage parametric-estimation mapping model (OCVPE) to reduce systematic errors of battery cells effectively within the temperature range of −20 °C to 40 °C. Although it has a high estimation accuracy, it takes a lengthy period of inactivity for the battery to attain thermodynamic equilibrium, making the OCV method inappropriate for real-time SOC measurements [7]. Therefore, direct measurement methods have limitations in measurement accuracy and the experimental environment, and they are less practical.

Model-based method predicts the SOC by combining filtering algorithms and observers to build a mathematical model [8,9]. Xiao et al. [10] used recursive least squares with a forgetting factor (FFRLS) to identify the model parameters, employed the adaptive extended kalman filter (AEKF) to identify the OCV and available capacity, and then input the estimated voltage and capacity into a second AEKF to achieve the estimation of the SOC. Gong et al. [11] proposed an improved OCV estimation method for batteries under low temperatures and adopted a dual adaptive extended kalman filter (DAEKF) based on the residual sequence to realize the simultaneous identification of model parameters and estimation of the SOC, achieving higher SOC estimation accuracy over a wide temperature range. However, the SOC is an internal state that is influenced by complex electrochemical dynamics. Under sustained low temperatures, these dynamics involve significant shifts in key parameters, complicating the task of creating accurate estimation for model-based methods.

Data-driven method collects a large amount of operational data of batteries under different working conditions, such as various currents, temperatures, and charge–discharge cycles; extracts characteristic signals including terminal voltage, current, temperature, and cumulative discharge capacity; and uses machine learning or deep learning models to establish a mapping relationship between input features and the SOC [12]. Data-driven methods provide a complementary approach by learning the mapping from operational data and applying it to the SOC. This paradigm is particularly suited for conditions like low temperatures, where deriving precise and adaptive physical models in real-time remains challenging. While traditional machine learning techniques have found broad application in SOC estimation, this approach offers a more robust alternative by lessening the dependency on predefined models [13,14,15]. Bobobee et al. [16] established an SOC estimation model with a temperature compensation function based on the particle swarm optimization (PSO) algorithm, realizing online diagnosis of lithium-ion battery states. To tackle the challenge of accurately detecting batteries across varying temperatures, they utilized a backpropagation neural network (BPNN) for a direct mapping between quantifiable battery metrics and the SOC, leading to a significant improvement in prediction accuracy. Deng et al. [17] proposed a data-driven method based on gaussian process regression (GPR). In addition to the conventional GPR model, they constructed an autoregressive GPR model to further improve the estimation accuracy and confidence and finally achieved relatively low estimation errors under extreme conditions.

In low-temperature environments, the electrochemical properties of batteries undergo significant changes. If the equivalent circuit model fails to accurately reflect the variations in these temperature-dependent parameters, such as ohmic internal resistance and polarization resistance, a large deviation will occur between the model’s output voltage and the actual terminal voltage, which, in turn, affects the state update and correction effects of the estimation algorithm, leading to an increase in SOC estimation error. In contrast, the time-series-based SOC prediction method can effectively address the characteristic of continuous changes in battery data under complex environments. Among these methods, long short-term memory (LSTM) networks [18] and their extended algorithms have demonstrated excellent performance. Liu et al. [19] extracted nine features from the current curves during the constant-voltage charging process and used Pearson’s correlation coefficients to select features. They proposed a multi-feature fusion model, which estimates the battery SOH by fusing different feature parameters and combining support vector regression (SVR) with LSTM networks. Chen et al. [20] proposed a novel long short-term memory recurrent neural network (LSTM-RNN) with extended input (EI) and constrained output (CO) for battery SOC estimation, which improves the SOC estimation performance.

The Bi-LSTM model is a variant of the LSTM model. It captures long-term temporal dependencies in two directions and can simultaneously process time-series information from both past and future directions [21]. With this feature, the Bi-LSTM model exhibits substantial enhancements in the precision of SOC forecasting [22]. In a recent study [23], a hybrid model combining a temperature-enhanced Bi-LSTM network and a multilayer perceptron (MLP) was proposed. The model is capable of adapting to SOC estimation under different temperature conditions. Bian et al. [24] used an encoder–decoder model combined with Bi-LSTM for SOC estimation under different temperature conditions. The method achieves a relatively low MAE under varying temperature conditions and enables effective monitoring of battery states in scenarios where the ambient temperature changes. Convolutional neural network (CNN) models are widely used as an effective means of extracting features from data. By combining the advantages of CNNs and Bi-LSTM, the hybrid model can effectively complete information extraction from lithium-ion battery data under various complex conditions. Reference [25] introduces a knowledge-constrained CNN-Bi-LSTM model (KCCL), which incorporates knowledge-based constraints during the training process to improve model performance under data scarcity conditions. Zafar et al. [26] developed a CNN-Bi-LSTM model based on fusion–fission optimization (FuFi) to evaluate the SOC at both high and low temperatures.

Since lithium-ion battery voltage, current, and temperature data in low-temperature environments are particularly susceptible to severe distortion, heightened noise interference, and complex nonlinear dynamics during collection and operation, the accurate estimation of the SOC becomes significantly more challenging. While various estimation paradigms exist, there remains a need for methods that are both robust and systematically validated for sustained operation in cold environments. To address these issues, this paper applies a hybrid CNN-Bi-LSTM-AM deep learning model for robust SOC estimation under low-temperature environments, with the following main contributions:

1. A hybrid deep learning architecture specifically tailored for low-temperature challenges is proposed. It synergistically integrates a 1D-CNN for extracting robust local features from noisy voltage and current sequences, Bi-LSTM for capturing complex bidirectional temporal dependencies, and an AM for dynamically weighting critical time steps [27], thereby enhancing model focus and suppressing interference from distorted signal segments.
2. A systematic and focused evaluation under persistent low-temperature conditions is conducted. Unlike studies that primarily test at room or broad temperature ranges, this work rigorously validates the proposed model across three specific low-temperature setpoints using standard driving cycles, explicitly demonstrating its robustness against temperature-induced nonlinearities and performance degradation.
3. Better estimation accuracy and comparative advantage are demonstrated. The model outperforms established benchmarks and other recent methods, confirming its effectiveness and advancement for low-temperature battery SOC estimation.

**Figure 1:**
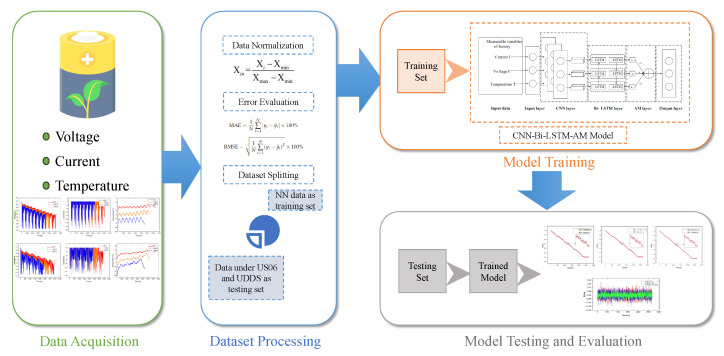
System framework diagram of lithium-ion battery SOC estimation.

The structure of this paper is outlined in the following manner: the design of the proposed CNN-Bi-LSTM-AM model is introduced in Section 2; the processes of dataset construction and processing are explained in Section 3; the results and analysis are discussed in Section 4; and finally, the conclusion based on the results and future work are provided in Section 5. Figure 1 shows the system framework diagram of this study.

## 2. CNN-Bi-LSTM-AM Model Design

### 2.1. Convolutional Neural Network

**Figure 2:**
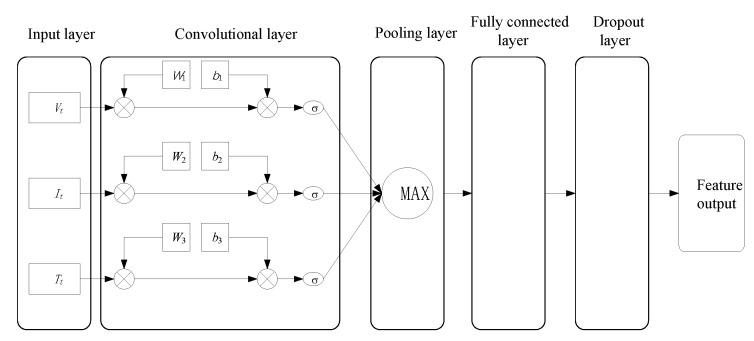
CNN model structure.

Yann and his team [28] introduced the concept of the CNN, a specialized type of deep learning network tailored for analyzing local patterns, like those found in images and sequences. For this particular investigation, the input features used in this study are the voltage, current, and temperature of battery data, all of which are time-series data. Hence, we applied the 1D-CNN to extract the SOC variation trend. The model structure of the CNN is shown in Figure 2.

The calculation formula of the CNN: if it is expressed in layers, the output of the first layer is

(1) $$X_{t}=\sigma \left(W_{t}*K_{t}+b_{t}\right)$$

In Equation (1), $K_{t}$ is the sequence data of the input layer at time *t* in Figure 2; $W_{t}$ and $b_{t}$ are the weight matrix and bias, respectively, which need to be trained to obtain the optimal parameters; σ is the activation function; ∗ represents discrete convolution; and $X_{t}$ is the output sequence.

Each convolutional layer operates by performing a one-dimensional convolution on the input data. Unlike autoencoder neural networks, these layers offer superior tracking capabilities and enhanced robustness, making them particularly effective at capturing the nuanced local nonlinear features inherent in the information [29].

### 2.2. Bi-LSTM Network

LSTM manages to retain long-term data and refresh short-term details by employing the gated structures, including the input, forget, and output gates, in conjunction with the cell states. Its structure includes the following elements:

(2) $$f_{t}=\sigma \left(W_{f}\cdot \left[h_{t-1},x_{t}\right]+b_{f}\right)$$

(3) $$i_{t}=\sigma \left(W_{i}\cdot \left[h_{t-1},x_{t}\right]+b_{i}\right)$$

(4) $$\tilde{C_{t}}=\tanh\left(W_{c}\cdot \left[h_{t-1},x_{t}\right]+b_{c}\right)$$

(5) $$o_{t}=\sigma \left(Wo\cdot \left[h_{t-1},x_{t}\right]+b_{o}\right)$$

In Equations (2)–(5), σ acts as the activation function; hyperbolic tangent serves as the tanh activation function; $h_{t-1}$ is the output at time t−1; $f_{t},i_{t},\tilde{C_{t}},o_{t}$ are the forget gate, input gate, candidate cell state, and output gate at time *t*; $W_{f},W_{i},W_{c},$ and $W_{O}$ are the weights corresponding to each layer; and $b_{f},b_{i},b_{c},$ and $b_{o}$ are the bias coefficients corresponding to each layer.

Finally, the current cell state $C_{t}$ is updated as

(6) $$C_{t}=f_{t}*C_{t-1}+i_{t}*{\tilde{C}}_{t}$$

**Figure 3:**
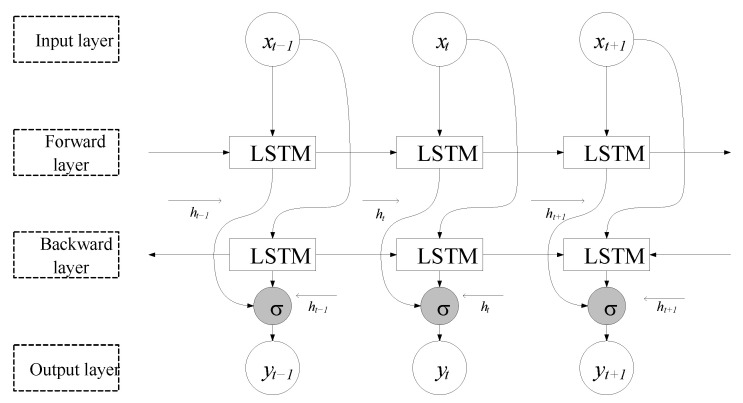
Basic structure of a Bi-LSTM model.

Bi-LSTM is an extension of the conventional recurrent neural network (RNN) architecture. Traditional RNNs lack the ability to solve long-term data dependencies during training [30]. Bi-LSTM integrates two separate LSTM networks, one that reads sequences from start to finish and another that processes them in reverse. This design allows the model to consider both the previous context and the upcoming context simultaneously, enhancing its capacity to handle time-dependent data effectively compared with single LSTM. Its structural composition is shown in Figure 3.

### 2.3. Attention Mechanism

The introduction of the AM is specifically motivated by the challenges of low-temperature SOC estimation. The AM enables models to efficiently capture long-range dependencies and has achieved success in time-series prediction [31]. Under low-temperature conditions, battery voltage profiles exhibit severe distortion and heightened noise, making it difficult for a model to discern which time steps are most indicative of the true SOC. A standard Bi-LSTM model processes all time steps equally, which can cause the influential signal from critical events to be diluted by less informative periods. The AM addresses this by learning to dynamically re-weight the output hidden states of the Bi-LSTM at each sequence step. It effectively acts as an adaptive filter, allowing the model to focus its capacity on segments of the data where the voltage–current dynamics are most strongly correlated with the SOC transition, thereby significantly improving estimation robustness and accuracy in the face of low-temperature nonlinearities. The specific implementation steps are as follows:

(7) $$s_{t}=\tanh\left(W_{h}h_{t}+b_{h}\right)$$

where $h_{t}$ is the hidden state of the input sequence at the *t*-th time step; $W_{h}$ is the weight matrix, which is used for the linear transformation of the hidden state $h_{t}$; $b_{h}$ is the bias vector; and $s_{t}$ is the score vector at the *t*-th time step. Subsequently, we apply the softmax function to normalize the input vectors and determine their respective weights $\alpha_{t}$.

(8) $$\alpha_{t}=\frac{\exp(s_{t}\cdot \nu )}{\sum_{t}\exp\left(s_{t}\cdot \nu \right)}$$

(9) $$h_{t}^{*}=\sum_{t=1}^{n}\alpha_{t}h$$

(10) $$y_{t}=\sigma \left(W_{y}h_{t}^{*}+b_{y}\right)$$

where $h_{t}^{*}$ is the weighted sum of the input hidden states, σ is an activation function, $W_{y}$ is the weight matrix, $b_{y}$ is the bias vector, and $y_{t}$ is the output at the *t*-th time step.

### 2.4. CNN-Bi-LSTM-AM Network

**Figure 4:**
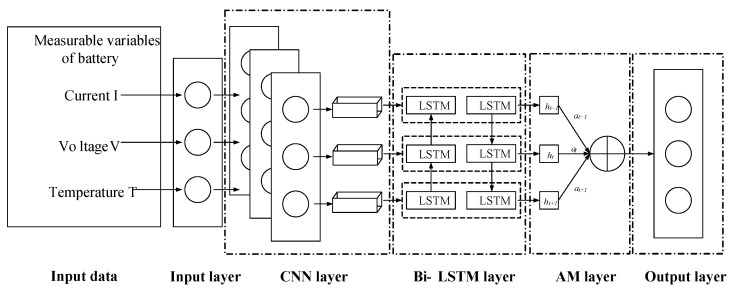
CNN-LSTM-AM framework.

The structure of the CNN-Bi-LSTM-AM model used in this paper is shown in Figure 4. The input layer receives key features, including voltage, current, and temperature. The CNN layer extracts features from the battery data. The Bi-LSTM network combines the forward LSTM and the backward LSTM and processes bidirectional time-series information simultaneously. The AM layer then assigns varying weights to each feature based on their significance, quantifies the correspondence between the predicted values and the time-series data, and determines the attention weights accordingly. Ultimately, the model outputs the estimated SOC value for the battery.

## 3. Dataset Construction and Processing

### 3.1. Dataset Construction

This study uses the publicly available Panasonic 18650PF battery dataset provided by the University of Wisconsin–Madison [32]. The dataset includes four car-driving conditions commonly used for SOC estimation, namely US06, UDDS, HWFET, and LA92, as well as a dedicated neural network (NN) driving cycle. The NN driving cycle is composed of partial data from battery cycling under the US06 and LA92 cycles, which are designed to have some additional dynamics useful for training neural networks. The experiments were conducted under constant ambient temperature conditions of −20 °C, −10 °C, and 0 °C, with the temperature held stable throughout each individual driving cycle. The discharge cycle of the dataset is collected at 0.1 Hz. The battery specifications and dataset information are summarized in Table 1.

To evaluate the SOC prediction performance of the CNN-Bi-LSTM-AM algorithm for lithium-ion batteries and considering that batteries are exposed to numerous complex operating environments under actual operating conditions, simulations of lithium-ion battery operating conditions are conducted under the US06 and UDDS driving cycles, respectively [34]. The input data (voltage, current, and temperature) collected under −20 °C, −10 °C, and 0 °C in these two driving cycles is used as an unknown data test set to assess the SOC estimation performance of the algorithm. Meanwhile, the NN data collected under −20 °C, −10 °C, and 0 °C is employed as the training set for training the machine learning model.

**Figure 5:**
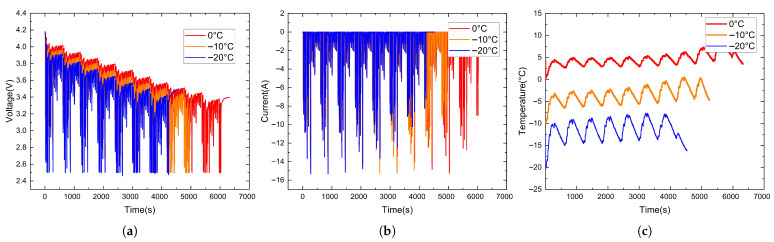
NN data collected at different temperatures. (**a**) Voltage variation curves. (**b**) Current variation curves. (**c**) Temperature variation curves.

**Figure 6:**
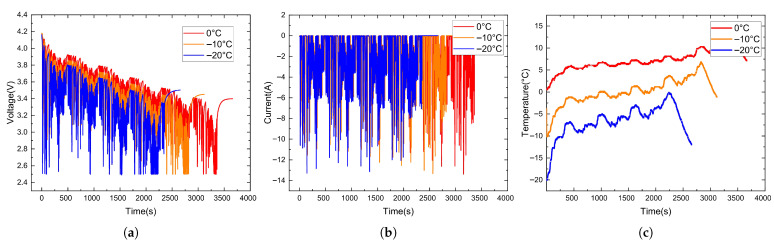
Data collected under the US06 driving cycle. (**a**) Voltage variation curves. (**b**) Current variation curves. (**c**) Temperature variation curves.

**Figure 7:**
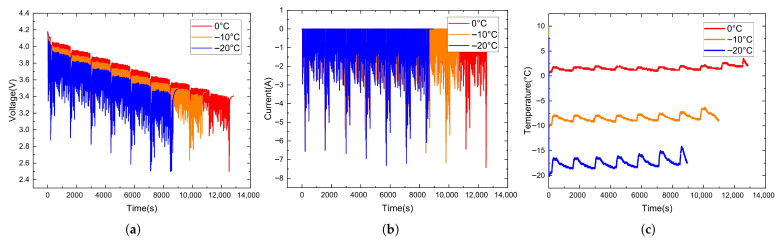
Data collected under the UDDS driving cycle. (**a**) Voltage variation curves. (**b**) Current variation curves. (**c**) Temperature variation curves.

The NN data collected at different temperatures are shown in Figure 5, and the data collected under the US06 and UDDS driving cycles are shown in Figure 6 and Figure 7.

### 3.2. Dataset Processing

#### 3.2.1. Data Normalization

To address the dimensional disparities among input features that may lead to a biased weight distribution during model training, Min-Max normalization is applied to scale the data to the range of [0, 1] [17]. Sequence-wise normalization rather than global normalization is employed to independently process voltage, current, and temperature sequences, which effectively prevents data leakage while preserving the intrinsic temporal dynamics of each physical variable with distinct characteristics. The equation is as follows:

(11) $$X_{\mathrm{in}}=\frac{X_{i}-X_{\min}}{X_{\max}-X_{\min}}$$

In Equation (11), $X_{i}$ and $X_{\mathrm{in}}$ represent the initial and scaled values, respectively, and $X_{\max}$ and $X_{\min}$ represent the sequence‘s maximum and minimum.

#### 3.2.2. Error Evaluation Index

This study assesses the SOC estimation accuracy of the proposed method by using the RMSE and MAE, which are defined as

(12) $$\mathrm{MAE}=\frac{1}{N}\sum_{i=1}^{N}\left|y_{i}-{\hat{y}}_{i}\right|\times 100\%$$

(13) $$\mathrm{RMSE}=\sqrt{\frac{1}{N}\sum_{i=1}^{N}{\left(y_{i}-{\hat{y}}_{i}\right)}^{2}}\times 100\%$$

In the equation, *N* denotes the complete set of samples; $y_{i}$ represents the authentic SOC value via Coulomb counting; and ${\hat{y}}_{i}$ indicates the approximated SOC figure from SOC estimation. The prediction performance is inversely proportional to the magnitudes of the MAE and RMSE.

## 4. Example Results and Analysis

### 4.1. Simulation Environment

**Figure 8:**
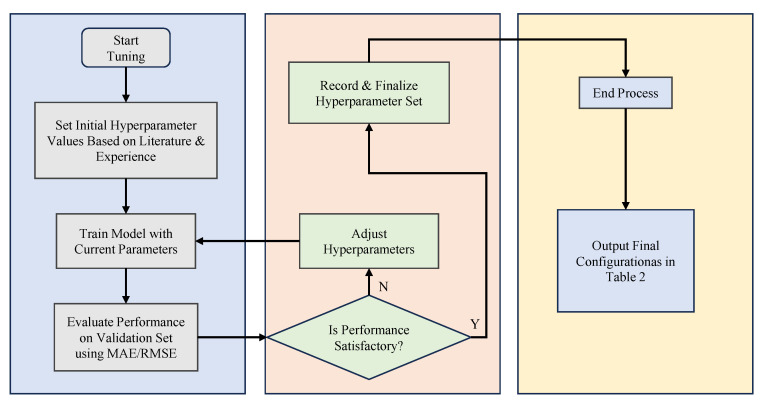
Hyperparameter tuning process.

The simulation environment of the study is as follows: CPU is an Intel(R)Core(TM)i7-9700 CPU@ 3.00 GHz; the operating system is Windows10; and the Matlab compilation environment is used. For the CNN part, a one-dimensional convolutional layer with 64 filters is adopted to extract local features. The dimensions of Bi-LSTM are all set to 64. For the AM part, the ReLU activation function and Sigmoid activation function are used after the first fully connected layer and the last fully connected layer, respectively. The Adam optimizer is used to train the model through iterative epochs, with an initial learning rate of 0.001, and the maximum number of iterations is set to 700. The mini-batch size is set to 64 to make the convergence more stable and accelerate the training speed. The MSE is used as the loss function and is defined as

(14) $$\mathrm{MSE}=\frac{1}{N}\sum_{i=1}^{N}{\left(y_{i}-{\hat{y}}_{i}\right)}^{2}\times 100\%$$

where *N* represents the total number of samples in the current batch (corresponding to the mini-batch size value), $y_{i}$ is the true value of the *i*-th sample, and ${\hat{y}}_{i}$ is the predicted value of the *i*-th sample. The hyperparameter tuning process is shown in Figure 8. The configuration of the model’s hyperparameters is shown in Table 2.

### 4.2. Battery SOC Estimation

**Figure 9:**
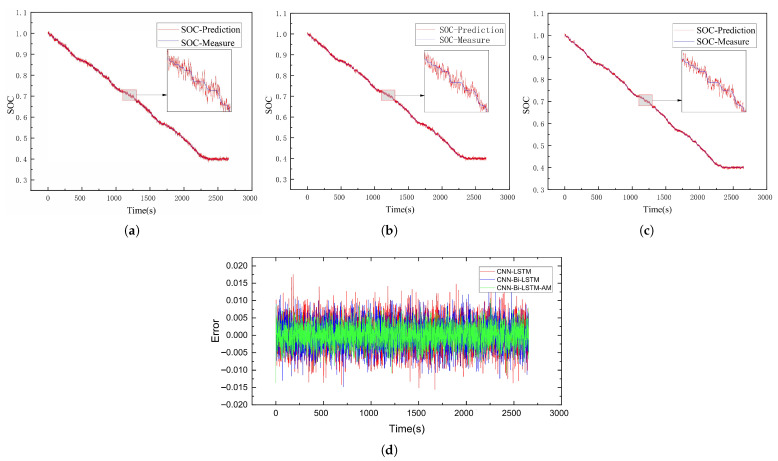
SOC prediction results of US06 at −20 °C using different algorithms. (**a**) CNN-LSTM. (**b**) CNN-Bi-LSTM. (**c**) CNN-Bi-LSTM-AM. (**d**) Error comparison.

**Figure 10:**
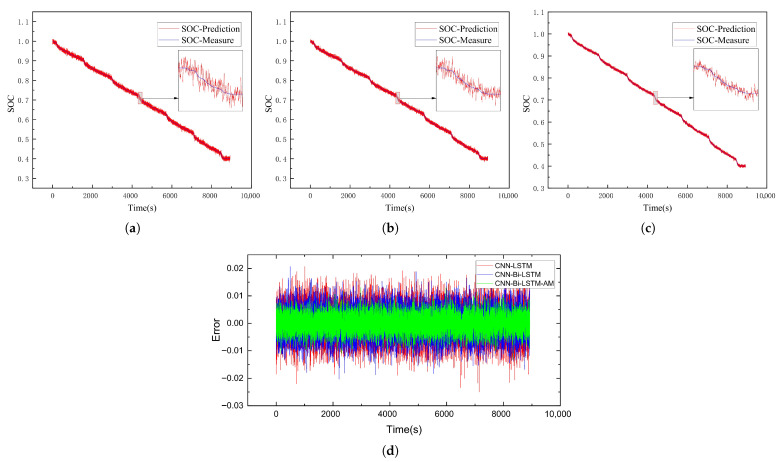
SOC prediction results of UDDS at −20 °C using different algorithms. (**a**) CNN-LSTM. (**b**) CNN-Bi-LSTM. (**c**) CNN-Bi-LSTM-AM. (**d**) Error comparison.

**Figure 11:**
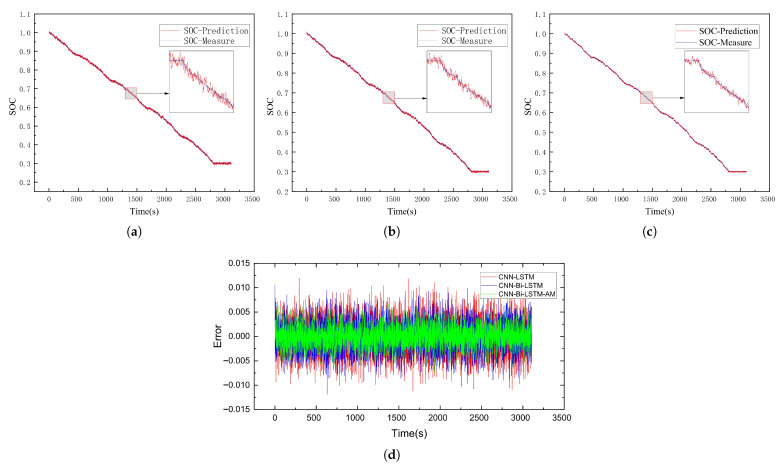
SOC prediction results of US06 at −10 °C using different algorithms. (**a**) CNN-LSTM. (**b**) CNN-Bi-LSTM. (**c**) CNN-Bi-LSTM-AM. (**d**) Error comparison.

**Figure 12:**
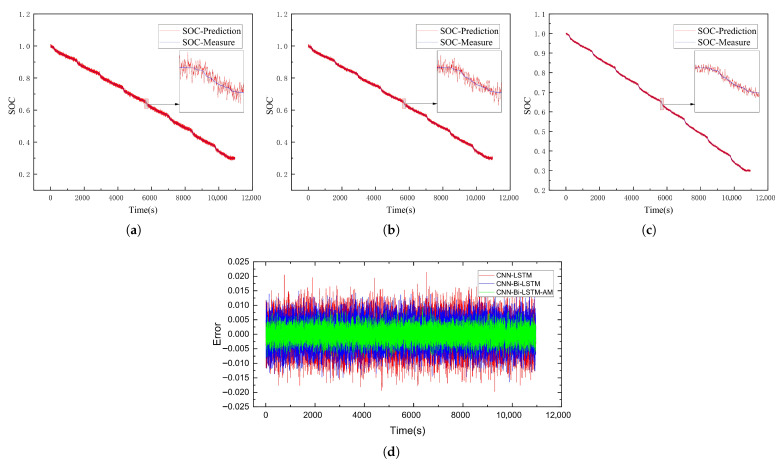
SOC prediction results of UDDS at −10 °C using different algorithms. (**a**) CNN-LSTM. (**b**) CNN-Bi-LSTM. (**c**) CNN-Bi-LSTM-AM. (**d**) Error comparison.

**Figure 13:**
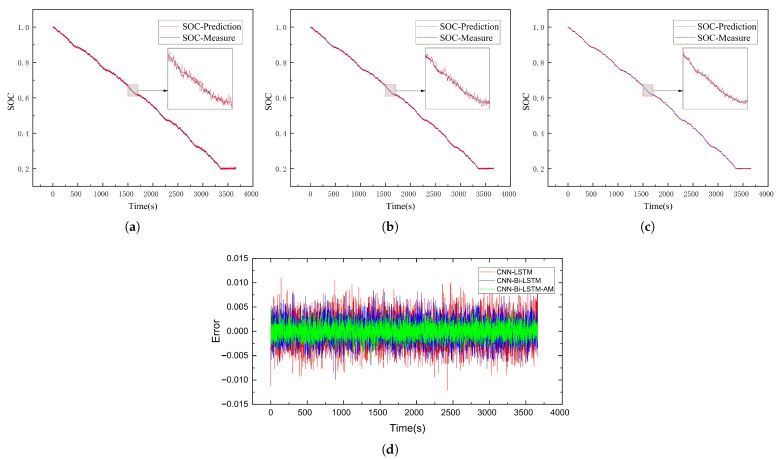
SOC prediction results of US06 at 0 °C using different algorithms. (**a**) CNN-LSTM. (**b**) CNN-Bi-LSTM. (**c**) CNN-Bi-LSTM-AM. (**d**) Error comparison.

**Figure 14:**
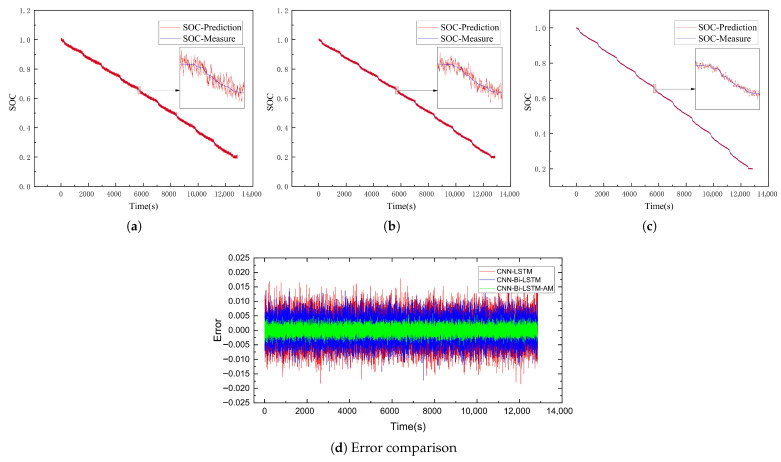
SOC prediction results of UDDS at 0 °C using different algorithms. (**a**) CNN-LSTM. (**b**) CNN-Bi-LSTM. (**c**) CNN-Bi-LSTM-AM. (**d**) Error comparison.

**Figure 15:**
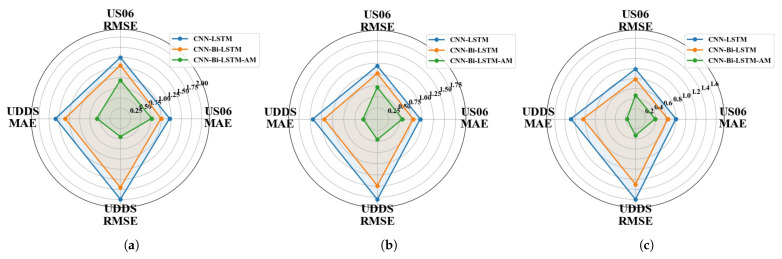
Model performance comparison at difference temperatures. (**a**) Comparison at −20 °C. (**b**) Comparison at −10 °C. (**c**) Comparison at 0 °C.

This subsection delves into evaluating the SOC estimation performance of the CNN-Bi-LSTM-AM model. To test its accuracy, battery data collected under the US06 and UDDS driving conditions served as test sets to assess the SOC prediction performance of the CNN-LSTM, CNN-Bi-LSTM, and CNN-Bi-LSTM-AM models at −20 °C, −10 °C, and 0 °C, respectively. The results of SOC predictions for each model are illustrated in Figure 9, Figure 10, Figure 11, Figure 12, Figure 13 and Figure 14. Additionally, Figure 15 summarizes the comparative performance of these different approaches in SOC estimation. The smaller the outline of the spider chart, the better the performance.

### 4.3. Analysis of Results

Based on the prediction results, the CNN-Bi-LSTM-AM model proposed in this paper achieves the best prediction performance under different temperatures for both driving cycles. In the harsh low-temperature environment of −20 °C, the estimation error of all models is the largest among all temperature gradients, indicating that low temperatures pose a significant challenge to battery SOC estimation. On the US06 dataset, the MAE of CNN-Bi-LSTM-AM is 0.77% and the RMSE is 0.94%, while the errors of the baseline models are approximately 57% and 60% higher, respectively. On the UDDS dataset, the errors are generally higher than those on the US06 dataset, primarily because the UDDS cycle’s frequent low-current fluctuations under low temperatures result in more subtle and noise-prone voltage dynamics, making SOC tracking more challenging compared to the high-current, more discernible dynamics of the US06 cycle. However, the CNN-Bi-LSTM-AM model shows even more remarkable advantages compared with CNN-LSTM, with its MAE reduced by approximately 63%.

As the temperature rises from −20 °C to −10 °C, the estimation errors of all models show a significant downward trend, indicating that the improvement in the battery’s operating state directly leads to an enhancement in model performance. On the US06 dataset, the average MAE of the three models decreases by approximately 23% compared to that at −20 °C. Among them, the MAE of CNN-Bi-LSTM-AM drops from 0.77% to 0.56%, with a reduction rate of 27%. On the UDDS dataset, the performance improvement is even more significant, with a reduction rate as high as 45%.

Under the environment of 0 °C, the battery’s performance further recovers, and the SOC estimation accuracy of all models reaches the optimal state. On the US06 dataset, the MAE and RMSE of CNN-Bi-LSTM-AM are as low as 0.39% and 0.47%, respectively; on the UDDS dataset, it performs even better, with an MAE of 0.17% and an RMSE of 0.33%.

Hybrid-structure models typically achieve better results in complex tasks [35]. Compared with the CNN-LSTM and CNN-Bi-LSTM benchmarks, the proposed CNN-Bi-LSTM-AM model demonstrates significant advantages in estimation accuracy under low-temperature conditions. Across all tested temperatures and driving cycles, CNN-Bi-LSTM-AM consistently achieves the lowest MAE and RMSE. The improvement is primarily attributed to the incorporation of the AM, which enables the model to dynamically focus on the most informative time steps while suppressing noise and distortion prevalent in cold-environment operation.

In addition to the two baseline models, the CNN-Bi-LSTM-AM model proposed in this paper also has greater advantages over other existing models. The performance metrics reported are derived from a direct comparison with several recent SOC estimation methods, as detailed in Table 3. The comparative analysis confirms the relative improvement offered by our proposed architecture within the domain, especially under high-dynamics profiles at low temperatures with an MAE of 0.17–0.77% and an RMSE of 0.33–0.94%, which indicates that the method this paper proposed has stronger capabilities in capturing complex nonlinear features, modeling long-term temporal dependencies, and highlighting key input features.

To evaluate the computational efficiency and embedded deployment potential of the proposed CNN-Bi-LSTM-AM model, we compare it against several benchmark and contemporary models [36] in terms of estimation time, parameter count, and model size, as summarized in Table 4.

The model achieves an inference time of 0.40 ms, which is competitive and suitable for real-time BMS applications. With 1.54 million parameters and a model size of 4.70 MB, the proposed model is more compact than Mamba and Kolmogorov–Arnold networks (KANs) and more capable than the simpler CNN-LSTM model. The profile confirms that the added AM provides significant accuracy gains under low-temperature conditions without incurring prohibitive computational overhead, supporting its potential for embedded deployment.

## 5. Conclusions and Future Work

### 5.1. Conclusions

This study addresses the challenge of accurate SOC estimation for lithium-ion batteries in low-temperature environments by applying a hybrid CNN-Bi-LSTM-AM model. By effectively extracting local features under noisy conditions, capturing bidirectional temporal dependencies, and dynamically focusing on critical time steps, the model significantly improves the accuracy of SOC estimation in low-temperature operation. Comprehensive comparative experiments demonstrate that the proposed model achieves relatively lower MAE and RMSE values under challenging low-temperature conditions, outperforming benchmark approaches as well as other referenced methods. This study presents a powerful and adaptable deep learning framework capable of effectively handling the severe nonlinearity and voltage distortion characteristic of low-temperature operation. The model’s robustness and adaptability under fluctuating low-temperature operating conditions offer a reliable solution for battery management in demanding scenarios.

### 5.2. Future Work

While this study has demonstrated the high accuracy and robustness of the proposed CNN-Bi-LSTM-AM model for SOC estimation under low-temperature discharge conditions, several avenues for future research are envisioned to advance this work towards real-world application.

1. The logical next step is to transition from simulation to embedded deployment. Future work will focus on porting and optimizing the trained model for execution on embedded BMS hardware.
2. To fully assess the model’s generalizability, evaluation under a complete spectrum of real-world operating conditions is essential.
3. Exploring lightweight and efficient data preprocessing techniques will be considered.

## Figures and Tables

**Table 1:** Battery specifications and dataset information.

Item	Specification
Cell Type	18650 Cylindrical
Chemistry	Panasonic NMC/Graphite
Nominal Capacity	2.9 Ah
Nominal Voltage	3.6 V
Voltage Range	2.5–4.2 V
Total Number of Cells	1
Operating Temperature	−20–60 °C
Test Temperatures	−20 °C, −10 °C, 0 °C
Data Source	Public dataset provided by the University of Wisconsin–Madison [33]

**Table 2:** Configuration of the model’s hyperparameters.

Hyperparameters	Value
Number of CNN filters	64
Number of hidden layer neurons	64
Excitation	ReLU, Sigmoid
Data sampling interval	0.1 s
Optimizer	Adam
Initial learning rate	0.001
Mini-batch size	64
Max epochs	700
Loss function	MSE

**Table 3:** Comparison of errors with other methods.

Reference	Method	Temperature (°C)	Performance
[6]	OCV-PE	−20, −10, 0	MAE = 4.1–4.9% RMSE = 2.32–3.31%
[10]	FFRLS-AEKF	0	MAE = 0.91% RMSE = 1.52%
[11]	OCV-DAKEF	−10, 0	RMSE = 0.65–0.86%
[17]	Autoregressive GPR	0	RMSE = 1.91–2.99%
[19]	EI-LSTM-CO	0	RMSE = 1.3–1.5%
[23]	Bi-LSTM encoder-decoder	−20, −10, 0	MAE = 1.26–2.32%
Our Study	CNN-Bi-LSTM-AM	−20, −10, 0	MAE = 0.17–0.77% RMSE = 0.33–0.94%

**Table 4:** Model computational load comparison.

Performance	Mamba	KAN	CNN-LSTM	CNN-Bi-LSTM	Proposed
Estimation Time (ms)	0.36	0.49	0.31	0.49	0.40
Params (M)	2.64	1.47	0.78	0.83	1.54
Model Size (MB)	10.56	5.88	2.49	2.66	4.70

## Data Availability

The original contributions presented in this study are included in the article. Further inquiries can be directed to the corresponding authors.
